# Lessons from conducting a participatory evaluation of a kinship navigator program

**DOI:** 10.1186/s40900-023-00487-6

**Published:** 2023-09-11

**Authors:** Erika Moldow, Virgie M. Anderson, Stephanie LaShay Benjamin, Barbara Patricia Johnson, Elizabeth McGuan, Donna Xenakis, Alexandra Piñeros Shields, Yanfeng Xu

**Affiliations:** 1 Elm Consulting, LLC, Denver, CO USA; 2 HALOS, Charleston, SC USA; 3 Heller School for Social Policy, Brandeis University, Waltham, MA USA; 4 College of Social Work, University of South Carolina, Columbia, SC USA

**Keywords:** Participatory evaluation, Program evaluation, Equitable evaluation, Kinship care, Kinship navigator program, Lived experience, Public involvement, Methodology

## Abstract

**Background:**

Approximately one in ten children globally live with kinship caregivers—relatives and family friends who step in to care for a child when parents are unable to do so. When families take on the role of informal kinship care—care of a child outside of the child welfare system—they often do so without financial assistance and advice in navigating the systems of support available to them. This is the unique role of kinship navigator programs in the U.S: to provide kinship caregivers a single point of entry for connecting to needed resources such as financial, health, housing, and legal assistance.

**Methods:**

To the best of our knowledge, our team conducted one of the only participatory evaluations in which kinship caregivers were involved in all stages of evaluating a kinship navigator program—from designing the questions, to collecting and analyzing the data, to reporting the results. Black kinship caregivers took on decision-making power leading this formative evaluation of a kinship navigator program within one nonprofit organization in a Southeastern state.

**Findings:**

In this paper we reflect on our process and offer lessons learned from engaging in participatory evaluation that may apply to the field of kinship care and across social service delivery more broadly. We focus on (1) ensuring the nonprofit’s commitment to the study, (2) maintaining engagement through building relationships and facilitating a culture of learning within the study team, (3) sharing decision-making power so that people with lived experience have the authority and ownership to lead the evaluation, (4) developing team members’ skills, confidence, and sense of belonging, and (5) increasing the likelihood the nonprofit will act on the study findings.

**Conclusion:**

Through this process, we learned that participatory evaluation is a feasible and useful approach both to understanding the experiences of kinship families and to improving the supports in their lives. We hope this paper will inspire others to draw on the strengths and capacity of people with lived experience to engage in participatory evaluation. Greater recognition of the value of this approach in social change and increased funding to carry out the process are both needed.

## Abstract (plain language summary)

Involving people with lived experience in all stages of an evaluation can strengthen the credibility of the findings. This paper provides an example of this involvement. It focuses on a program designed to support kinship caregivers—those who step in to take care of a child when the parents are unable to do so. To the best of our knowledge, our team conducted one of the only participatory evaluations in which kinship caregivers were involved in all stages of evaluating a kinship navigator program in the U.S.—from designing the questions, to collecting and analyzing the data, to reporting the results. Through this process, we learned that participatory evaluation is a feasible and useful approach both to understanding the experiences of kinship families and to improving the supports in their lives. In this paper we share our collaborative journey through each stage of the evaluation, offering lessons learned about the process that may apply to the field of kinship care as well as to other areas of social service delivery.

## Introduction

Participatory evaluation is rooted in the belief that involving people with lived experience in all stages of a program evaluation can promote a more inclusive approach to organizational learning and lead to more credible and useful findings [1, 2]. This paper shares an example of this involvement within the field of kinship care. It focuses on a program designed to support kinship caregivers—those who step in to take care of a child when parents are unable to do so. In this commentary, we describe how Black kinship caregivers on the evaluation team stepped in to lead each stage of a program evaluation. We reflect on our process and offer lessons learned from our participatory approach that may apply to the field of kinship care and across social service delivery more broadly.

## Background

Approximately one in ten children globally live with kinship caregivers—relatives and family friends who step in to care for a child when parents are unable to do so [3]. Children in kinship care are more likely to have faced childhood trauma and to have more significant mental health needs than children living with a biological parent [4, 5]. When families take on the role of informal kinship care—care of a child outside of the child welfare system—they often do so without financial support and assistance navigating the systems of support available to them [6]. This is the unique role of kinship navigator programs in the U.S.: to provide kinship caregivers a single point of entry for connecting to financial, health, housing, and legal assistance, as well as other supports for the safety and well-being of children in their care.

To build effective programs that support kinship families, caregiver voice is essential [7]. There are prominent examples of kinship caregivers stepping into positions of power, using their voice as strategic advisors, advocates, staff, and peer mentors [8–11]. To the best of our knowledge, our team conducted one of the only studies engaging kinship caregivers in all stages of evaluating a kinship navigator program in the U.S.—from designing the questions, to collecting and analyzing the data, to reporting the findings.

Public involvement in research recognizes the importance of studies being carried out “with” or “by” the public rather than “about” or “for” them [12]. There are many terms to describe public involvement in research globally. Our approach is most closely aligned with the structure of a participatory evaluation. Participatory evaluation is increasingly being recognized as an effective approach to gathering valid, culturally relevant data across diverse fields [13]. In one stream of this approach—practical participatory evaluation—program recipients, who often lack a voice in program and policy decisions, can influence program decision making [1, 14]. This approach has roots in the U.S. and Canada dating back to the 1970s with the goal of enhancing evaluation ownership and use [14]. The structure often involves a collaborative partnership between stakeholders who hold intimate knowledge of the context, organization members who have a role in running a program, and a trained evaluator [1, 15].

This participatory approach advances culturally responsive evaluation, deeply engaging historically marginalized groups in the evaluation process [16]. The more involved stakeholders are in the central tasks of the evaluation, the more participatory the evaluation is considered to be [17]. Using a participatory approach, the power relationship between social service providers and clients is flipped, allowing program recipients to step into their power by evaluating the performance of social service delivery [18].

There is a need to involve people with lived experience in determining the supports that would make a difference in their lives. “People with lived experience” refers to:

individuals directly impacted by a social issue or combination of issues who share similar experiences or backgrounds and can bring the insights of their experience to inform and enhance systems, research, policies, practices, and programs that aim to address the issue or issues [19].

By involving people with lived experience, evaluations can then benefit from a deeper understanding of the conditions in which families live and the supports that are most needed [19]. Moreover, ensuring that Black kinship caregivers have decision-making power at every stage of an evaluation is an important move towards racially equitable evaluation methods [20]. The Center for Evaluation Innovation [20] defines equitable evaluation as:

Aligning our practices with an equity approach—and even more powerfully, using evaluation as a tool for advancing equity. It means considering these four aspects, all at once:

1. Diversity of our teams (beyond ethnic and cultural)
2. Cultural appropriateness and validity of our methods
3. Ability of our designs to reveal structural and systems-level drivers of inequity
4. Degree to which those affected by what is being evaluated have the power to shape and own how evaluation happens.

Chukwudozie et al. [21] conducted one of the few published studies engaging kinship caregivers as co-researchers through all stages of their research exploring informal kinship care practices in 17 communities across seven states/provinces in West and Central Africa. This study conducted in Africa shows both the value of involving local researchers who understand the context of kinship care and the importance of ongoing training in data analysis and reporting to ensure study rigor.

## Aims and rationale

This participatory evaluation was designed to gather data within a nonprofit organization in a Southeastern state in the U.S. serving kinship families, to strengthen the program model and service delivery. Formative assessments are critical in providing immediate and concrete suggestions to improve the program and ensure services are tailored to the unique needs of the population before engaging in a summative evaluation [22]. Further, the literature strongly supports involving people with lived experience in deep, meaningful ways in the very programs designed to meet their needs [7, 21]. The findings from our evaluation can be found in a separate evaluation report [23]. In this paper we reflect on our participatory process and offer lessons learned from conducting a formative evaluation that may apply to the field of kinship care and across social service delivery. Our evaluation report and this commentary were co-authored by the caregivers on our study team.

## Context of the evaluation

To offer important context, the following section briefly describes kinship care and the kinship navigator program that is the focus of this evaluation.

### Kinship care

Kinship care is the “full-time care, nurturing, and protection of a child by relatives, members of their tribe or clan, godparents, stepparents, or other adults who have a family relationship to a child” [24]. We use the term “kin” and “kinship” interchangeably. Across the U.S., more than 2.5 million children are being raised in the homes of grandparents or other relatives [25]. Before the age of 18, 1 in 11 of all children and 1 in 5 Black children live at some point in kinship care [26].

Kinship care has historical roots in Black, Latinx, Asian, and Native American communities, keeping cultural identity and traditions alive, while protecting children from the trauma of entering the child welfare system [27–29]. Compared with non-kin foster parents, kinship caregivers tend to be older, lower income, and in poorer health [30, 31]. Despite these challenges, research shows that children fare better when placed in kinship care rather than in non-kin foster care [32].

This evaluation took place early in the COVID-19 pandemic when restrictions were high, with many kin caregivers experiencing increased economic hardship, parenting stress, and psychological distress [33, 34]. COVID brought unique challenges to all families, including kinship families, due to the closure of schools and daycare centers and the move to online learning, which resulted in an increased caregiving burden [35]. At the same time as kinship families faced greater needs, the availability of services, including health and mental health services, decreased [34].

#### Kinship navigator programs

In response to caregivers’ needs for connecting to financial, health, housing, and legal systems, kinship navigator programs have emerged across the U.S. Today, approximately 70 kinship navigator programs exist to support kinship families across 26 states in the U.S. [6]. Through support of the kin caregiver, these programs help ensure the safety, permanency, and well-being of children in the home. One kinship navigator program, typical of many programs emerging across the U.S. that provide a single point of contact for kinship families, is the HALOS Kinship Navigator Program, the focus of this evaluation.

### HALOS kinship navigator program

HALOS started as a nonprofit in 1997 and launched the first kinship navigator program in South Carolina in 2007. Program staff assist kin caregivers with understanding, navigating, and accessing the system of out-of-home care supports and services over a 3-month period. Most kin caregivers are informal caregivers, meaning that they are not formally connected to the child welfare system. During COVID, HALOS remained open, though some in-person supports became virtual and other resources were temporarily suspended.

During the focus of our study, March 2020 through February 2021, the program reached 146 kin caregivers and 479 kin children. The majority of kin caregivers were female (95%), grandparents (68%) and Black (59%). Of the 146, 30% reported having a disability and 77% had an annual income under $30,000, which is now the federal poverty level for a family of four [36].

## Evaluation approach

In the summer of 2020, HALOS Executive Director, Kim Clifton, and Kinship Navigation Director, Elizabeth McGuan, reached out to a community-based researcher on this team to initiate a formative evaluation to explore caregivers’ experience in the program and identify areas of program improvement. The community-based researcher was experienced in participatory evaluation, but new to the field of kinship care. We understood from the start that kin caregivers are experts who would be critical in designing the evaluation. In August 2020, HALOS invited a representative group of eight kin caregivers who had completed the 3-month kinship navigation program to join as advisors. The group included both new and experienced caregivers, diverse across gender, race, and age. We knew it was important to involve caregivers who were diverse not just in their personal characteristics, but also in the ways by which kin children came into their care, in their relationships to the kin children, and in their connections to the child welfare system. Cousins and Chouinard [1] find that diverse stakeholders can “bring a detailed and rich understanding of the community and program context” (p.5) and “can lead to more credible and valid evaluation findings” (p.149–150).

During the initial 2-month period, kin caregivers in our advisory group shared their own kinship stories and their experiences as participants in the program and at the same time learned about all aspects of the program model. We realized from these conversations that caregivers had strong qualitative research skills—strong listening skills, ease in building rapport, comfort in handling ambiguity, and patience in analyzing data. The next step was to move beyond an advisory role and ask kin caregivers of their interest in becoming core members of the evaluation team. After these first two months, we invited the five caregivers who remained in the group to join; ultimately, four signed on. Three of the caregivers were female, one was male, all were Black, and all were between the ages of 40 and 74. The four kin caregivers were helping to raise nine children ranging from 4 to 14 years of age.

By October 2020, the evaluation team of seven was complete (with study team initials listed in parentheses): Four kin caregivers (VMA, BPJ, SLB, CL), two staff members—the Kinship Navigation Director (EMcG) and Support Group Facilitator (DX)—and one outside evaluator (the first author) to guide the process (hereafter referred to as “the evaluation team” or “we”). Two academic advisors also supported our team, one a scholar in participatory action research (APS) and the other a statistician and scholar in kinship care (YX).

Together, we determined the evaluation questions:

1. How are kin caregivers adjusting to life with a kin child? What supports and resources are most needed, especially during the pandemic?
2. What are kin caregiver perceptions of the program? What are the program strengths and areas of improvement?
3. What changes do kin caregivers experience during their time in the program, particularly in their well-being and ability to meet financial needs?

## Methods

**Fig. 1:**
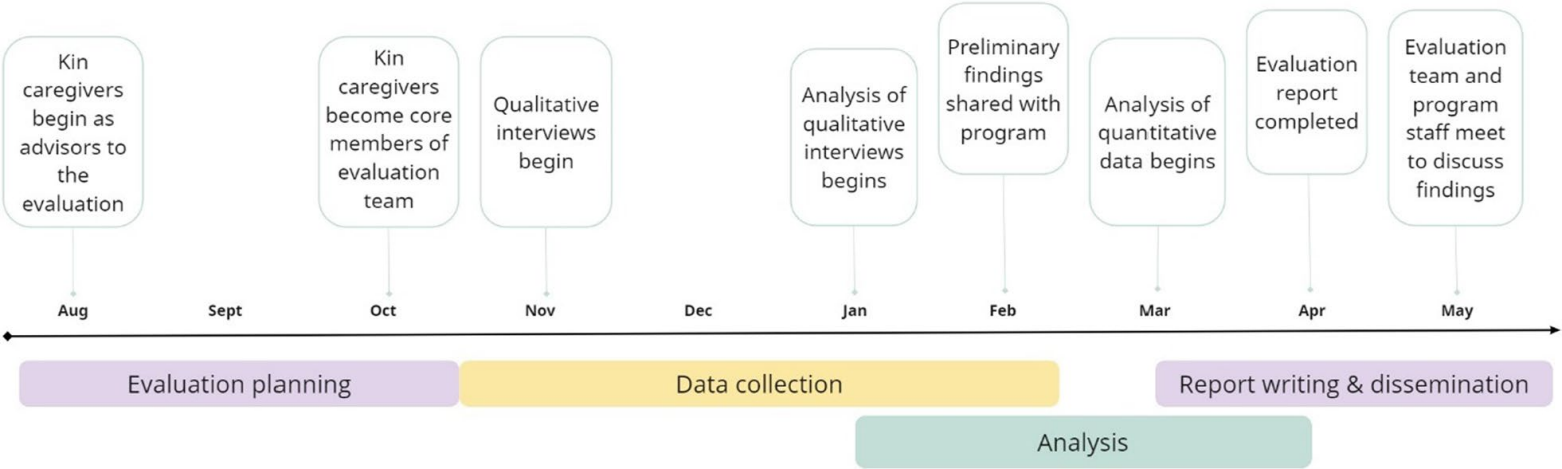
Participatory evaluation timeline

A participatory evaluation approach was used throughout the evaluation process, bringing together both qualitative and quantitative data. Our evaluation relied on primary data collected through caregiver interviews at program completion. Interviews were supplemented by secondary data collected by program staff at intake and at program completion. Measures are described below and can be found in detail in the full evaluation report [23]. The Colorado Multiple Institutional Review Board confirmed that this evaluation did not meet the definition of research under 45 CFR 46.102, and thus did not require approval or oversight. As this commentary describes the process of our evaluation, it does not meet the criteria for protection of human subjects. Figure 1 below presents a timeline of our process.

### Qualitative method

Our study team selected individual interviews as the primary methodology to learn more about caregiver experiences in the program. We decided that the kin caregivers on our team (VMA, BPJ, SLB, CL) would design the interview guide, conduct interviews, and interpret the data. To prepare for this new role, we met for nine hours to go through an interactive virtual training session led by the outside evaluator on our team. The training focused on the key concepts within the Collaborative Institutional Training Institute (CITI Program) Social and Behavioral Research Course [37]. This initial training covered key concepts of research involving human subjects—including confidentiality and privacy; risks and benefits to participants; voluntary participation; and dignity of persons—and qualitative interviewing.

Caregivers on our team generated interview questions using a process developed by the Right Question Institute [38]. Using their Question Formulation Technique, caregivers began by asking as many questions as they could, without discussion or judgment, and then prioritized what questions were essential to ask. For the interview, we decided to begin by asking interviewees about the number of children in their care as well as children’s ages and how they were related. We then asked how “settled” and “comfortable” everyone was in the family to allow caregivers the opportunity to share as much as they were willing. Knowing that caregiving could be demanding, we wanted to know how it felt for each of them on a personal level. Most of the interview focused on caregivers’ experience and satisfaction with the kinship navigator program. We asked why they connected to the program, what services and resources they took advantage of, and whether the program had met their needs. We wanted to know how, if at all, the program had impacted their lives. We then piloted the guide and made final changes.

#### Recruitment

All kin caregivers who started the kinship navigator program between June and September of 2020 were invited to take part in interviews after they finished the program. We first sent an introductory letter and consent form by mail and email. After a few days, we called to obtain informed consent by explaining the purpose of the evaluation, the voluntary nature of participation, and the risks and benefits of participation. We discussed the types of questions to expect during the interview and how their responses would be kept confidential. Caregivers received a $15 gift card for their participation. From the start we believed that caregivers were more likely to be at home and available for interviews during COVID. Of the 53 caregivers we contacted, 58% (N = 31) consented and were interviewed. Of the 53, 17% did not respond to our requests, 13% were not available during the scheduled interview time, 6% had non-working phone numbers, and 6% refused. We found that the population interviewed were similar to the population of HALOS caregivers across demographic characteristics. The Support Group Facilitator on our team (DX) oversaw all recruitment and scheduling.

#### Data collection

Between November 2020 and February 2021, the caregivers on our evaluation team conducted 31 interviews with kin caregivers once they finished the 3-month program. All interviews were semi-structured and conducted one-on-one, via phone. Most interviews were 15–25 min in length, with an average of 22 min. All were audio-recorded and transcribed by a professional transcription service. Caregivers on the team set the tone of each interview from the beginning by sharing a few personal details of their lives, immediately putting interviewees at ease and creating a safe, sharing environment. Once an interviewee discovered they had something in common, we heard a variety of positive reactions such as, “Wow, you’re a caregiver, too?” and (in response to the grandmother on our team raising five grandchildren), “Oh my, five children?!” The interview then benefited from being “a conversation between equals than as a distinctly hierarchical, question-and-answer exchange” [39].

During interviews, we discovered that kin caregivers were open to talking with their peers—sharing their lives, their vulnerabilities, and their ongoing needs. The caregivers on the team all had first-hand experiences with the child welfare system and knew by asking non-scripted follow-up questions they would build trust and give interviewees the sense they were connecting with someone who understood their journey. This approach recognizes the long history of distrust among families with the child welfare system, particularly among communities of color [40, 41].

#### Data analysis

After each interview, kin caregivers recorded their reflections as analytic memos [42]. In some cases, interviewers talked directly into a recorder, in most cases they called the evaluator or another member of the team to process their interview experience. The evaluation team joined in ongoing virtual meetings every week, often twice a week, for 90 min at a time to discuss the interview experience and to analyze each interview as a team. For each interview, and for every question in our guide, we listened to the response and read the interview text at the same time. Central guiding questions for us included: What did the kin caregiver say? What is important about their response? What did you learn? We recorded and transcribed all analysis meetings.

Over 8 months, we met for more than 50 h. Kin caregivers rarely missed a meeting, even attending from doctor’s offices, between work shifts, and while still recovering from COVID. This consistency helped us keep our momentum during analysis. Themes emerged inductively after careful review of every interview [43]. A data matrix helped us in comparing and contrasting responses to interview questions, following the advice of Miles, Huberman, and Saldaña [42].

To guide our analysis and strengthen the validity of our findings, we followed guidance from Maxwell [44]. We looked for patterns when additional information was consistent, but also for times when experiences diverged. In particular, we looked for examples of when caregivers’ needs were not met and why. We audio-recorded and transcribed all interviews to review as a team and thus to avoid interpreting an interview through the lens of any one person’s experience. During our team conversations, we talked not only about the interviews, but also about what the interviews brought up in each of us—to be up front with our own lenses and biases. We found that by the end of our 31 interviews we had reached saturation as many of the same themes were emerging.

### Quantitative method

To supplement caregiver interviews, we examined data collected by program staff at intake and at the end of the 3-month program. The program measured change in the adequacy of family resources, from basic necessities such as housing and food, to funds for bills, to time for family, through the Family Resource Scale [45]. In addition, the program also collected data on change in financial benefits (type of benefit, status of application, monthly sum received) and child stability (whether kin children have left the household at the end of the program). Of the 146 kin caregivers enrolled during the study period, 46 (32%) completed the 3-month follow-up. The statistician on our team (YX) ran tests of reliability for the overall scale and subscales of the Family Resource Scale, compared the demographics of the population who responded to the pre and post survey with the full population of HALOS caregivers, and analyzed change over time.

Our full evaluation team examined the survey results. Once our statistician completed analyses, she brought aggregate data, without names or identifiers, to three separate group meetings to ask kin caregivers on our team to reflect on the findings together. She asked kin caregivers for their interpretation of the data and how the data gathered through the program were consistent or different from the data gathered through interviews. When survey data showed that access to certain benefits was lower or higher than expected, kin caregivers on the team offered important context that might explain each finding, including information gathered from interviews and from their lived experience.

### Dissemination activities

**Fig. 2:**
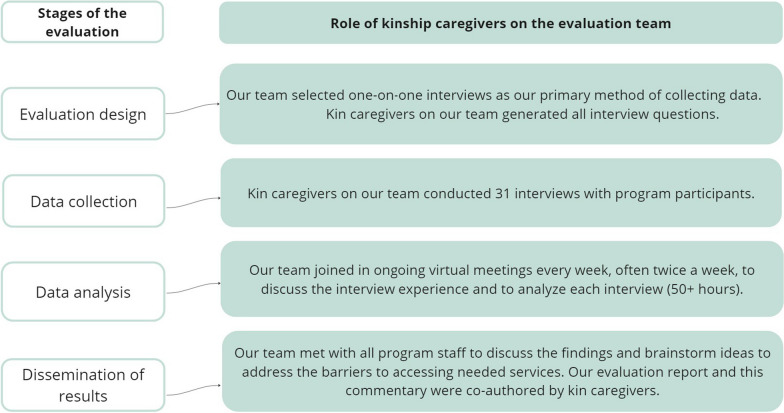
Role of kinship caregivers throughout the evaluation

Our team shared findings with HALOS program staff iteratively to avoid surprises and develop a better understanding of the types of challenges staff were facing. At the end of the evaluation, the team met with all HALOS staff to discuss the findings and brainstorm ideas to address the barriers to accessing needed services. Our team posed a series of questions for reflection, both in writing and in conversation, rather than a fixed set of recommendations. We did this realizing that we did not know all the dynamics involved in decision-making at the program level. The main themes and transcripts from our months of analysis meetings served as the text for our final evaluation report. Our evaluation team and advisors all co-authored the final report. In the end, a graphic designer, Oliver Moldow, joined several virtual calls to ask our team for advice in the report design. Figure 2 below offers an overview of the role of kinship caregivers throughout the study, from designing the evaluation through disseminating the results.

## Findings: lessons learned

Through our evaluation, we learned about the varied ways the kinship navigator program made a difference in the lives of kinship families. We also uncovered vital new information to help the program improve. Full findings can be found in our evaluation report [23]. In this commentary, we focus exclusively on the *process* of conducting a participatory evaluation and offer lessons learned that may apply to the field of kinship care and across social service delivery more broadly.

Overall, we learned that participatory evaluation is a feasible and useful approach both to understanding the experiences of kinship families and to improving the supports in their lives. Three aspects of our process are rare in participatory evaluation. First, Black kin caregivers, including Black grandparents, led all stages of a formative evaluation in a field in which they are the experts—an underutilized strategy in kinship care and in the wider field of child welfare [46]. Second, there was a depth of participation among kin caregivers throughout the evaluation, particularly throughout the analysis phase when study teams often see a drop-off in participation [2, 21]. Finally, HALOS, the nonprofit organization and focus of this study, showed ongoing commitment to the participatory process by valuing caregiver voice through all stages of the evaluation and immediately acting on the study findings—a welcome outcome considering that most evaluation findings are not utilized by programs [15].

In this section we share key lessons learned that fostered these successes. As there is no single method of conducting a participatory evaluation, we offer lessons from our experience. We audio-recorded and transcribed our meetings to reflect on our journey as a team, consistent with the longstanding practice of reflexivity in qualitative research [47] and consistent with Staley and Barron’s [48] approach to sharing researchers’ personal accounts in the service of learning. Our conversations took place over more than 50 h, through all stages of the evaluation. We share the words of the four kin caregivers on our evaluation team throughout this section to illustrate five key lessons.

### Lessons learned #1: ensuring the nonprofit’s commitment to the study

Belief in the importance of a participatory approach started at the top of the organization, with the HALOS Executive Director. There was an organizational commitment to the idea that those with lived experience have a critical role in producing knowledge for program improvement. Participatory evaluation as a method often requires a greater investment of time, program resources and organizational commitment [15]. In our experience, each stage of the evaluation, particularly managing computer issues and reviewing interview text virtually, consumed more time than expected. The funder offered flexibility to HALOS to allocate funds for their evaluation efforts in any way that would help them achieve their organizational goals.

HALOS prioritized the evaluation process, dedicating the necessary funds and appointing program staff to see the process through. From the outset, the Kinship Navigation Director invested time responding to any questions kin caregivers had about the program model and delivery. She voiced from the beginning and throughout the process that honest critiques are essential in improving the program. The Support Group Facilitator had years of experience running kinship support groups and was able to oversee the interviewee recruitment and consent process.

Participatory studies have lost local researchers due to lack of compensation [21]. No costs were absorbed by caregivers to carry out the work as Chromebooks, WiFi Boosters, and audio recorders were provided. On our team, caregivers were paid for their time—for every interview and meeting, for piloting interviews, for creating memos, for reviewing report drafts and for practicing the presentation. This flexible part-time work allowed caregivers to earn extra needed money when it fit best into their lives.

### Lessons learned #2: maintaining kinship caregiver engagement

Cousins and Chouinard [1] reveal that many participatory evaluations find it extraordinarily difficult to maintain stakeholder commitment for the duration of the process. During our initial 2-month period, four of the eight caregivers self-selected out. Initially this dropout was worrisome, leading to uncertainty as to whether the remaining caregivers would be able to continue. In hindsight, this introductory period was pivotal in identifying four kin caregivers who could commit to the process. The quality of their participation outweighed the attrition of the four other members. The remaining caregivers stayed throughout the 8-month evaluation, attending virtual meetings 1–2 times per week for 90 minutes.

Meetings and interviews were held around caregiver schedules, changing week to week to accommodate family sickness, work and family commitments, and life changes. We established a general timeline from the beginning but worked at a pace that the team could accommodate. Several strategies fostered kin caregivers’ ongoing commitment:

* Time for relationship building as a study team* The caregivers on our team bonded over their shared journey as well as their shared strength as deeply religious individuals. Historically, Black families turn to the church in distress for strength [49]. In the early planning stage, kin caregivers shared: “I feel the confidence. I love the people. I love y’all. We’re becomin’ a family,” and adding, “We all have something in common. It’s like therapy. Even though it’s helping the children, it’s helping us.” Caregivers on the team mentioned they looked forward to our time together even more because they had lost other connections in their lives during COVID.

* Culture of learning as a study team* For kin caregivers, the process of conducting an evaluation was novel. From the start, we built a culture of learning, knowing that we would make mistakes and learn along the way. Cousins and Earl [15] find that stakeholders engaged in a participatory process must have a “tolerance for imperfection” (p. 414). All kin caregivers on the team expressed nervousness before conducting their first interview. One member of our team noticed a change in her perspective even after the first few interviews: “I’m more relaxed now and I’m going with the flow. I feel so much better now when I do the interview. … I always learn something from each interview for the next interview.” Meetings served as a safe space for caregivers to share their insights, challenges, and mistakes, so we would all learn from each new interview. Engaging in the construction of meaning and knowledge invited caregivers to step into their power [50].

* Time for reflection as a study team* One of the challenges for caregivers on our team was in listening to caregiver stories that often mirrored their own life experiences. After a difficult first interview, one member of our team reflected: “You already went through some of the same things they are going through. That was hard for me. Afterwards, oh, my goodness, the burden was still on me. I said, ‘Oh, it was so hard. I have to talk to someone.’” After each interview, kin caregivers reached out to another team member, often the outside evaluator, to process their experience.

As a study team, we also made the decision early on to listen to each interview and read the interview text together at the same time, knowing we all learn differently. However, the act of listening to the interviews together became important in yet another way: it allowed all of us to share in the experience of the interviewer, to better understand how the interview felt, and to be a source of support to the interviewer. In one example, a caregiver praised another member of the team after listening to her emotional interview: “I feel a weight from this one. I don't know if you felt it when you did it, but you did really good. I'm really proud of you. It's just like a conversation between two friends, a friend you haven't talked to in a while, you're checking on her, and I love that.” Maintaining time and space for reflection enabled team members to examine their own experiences in a group setting to engage in deeper analysis of the data [51].

### Lessons learned #3: Sharing decision-making power

Bringing together program recipients, staff, and an outside evaluator requires attention to inherent power imbalances [16, 52]. According to Gaventa’s theory of power [18], this evaluation created an opportunity for program recipients to move from the first level of power, that of access, to the second level of power, influence over institutional decision-making. In our evaluation, we found that power could be distributed across the team due to a strong sense of authority and ownership kin caregivers established early on.

*(1) Authority to make decisions* Consistent with a central element of participatory work, our team had the “authority” to make decisions [16]. We valued each team member’s input, and all stages of the evaluation were based on group decision making, a practice of successful collaborations [53]. Hicks et al. [53] explain:

Stakeholders perceive a process as authentic when their input is valued and they believe that the collaborative process itself, rather than some external agency, has the authority to generate plans and have those plans guide decision making (p. 470).

After the initial 2-month period, when we asked caregivers if they were interested in becoming core members of the evaluation team, we heard comments such as: “It’s an honor to feel that my input matters and makes a difference,” and “It’s great to be heard and included.”

*(2) Ownership at each stage of the evaluation* Following advice from Cousins and Earl [15], we selected a methodology that caregivers could learn quickly and implement well. Caregivers were well positioned to make decisions—in design, analysis, and reporting—based on their lived experience as kin caregivers and their sense of ownership of the process. With training in interviewing as a methodology and time piloting the interview guide at the onset of the evaluation, caregivers developed confidence in their ability to lead the work. When we asked caregivers on the team why they remained on the team, we heard: “It’s a sense of purpose. I feel like I have something to contribute. I was honored to be asked. I feel like God is in the mix.” Another caregiver, who always livened up our meetings with his humor, responded, “You said there would be donuts,” and then continued with, “This is something I can be doing. … It’s very positive.” Caregivers on the team were motivated to continue, knowing that their contribution was helping HALOS improve and, in turn, helping other caregivers. In a genuine display of ownership, one kin caregiver on our team kept our evaluation report at her front door to show it to family members who visited.

### Lessons learned #4: developing team members’ skills, confidence, and sense of belonging

Through each stage of the evaluation, we could see changes in all of us. Participatory evaluations are linked to positive changes among stakeholders, including increased learning/capacity, awareness of issues, and confidence [1]. In particular, caregivers on our team came to see themselves as better listeners, more confident, more knowledgeable about their rights in the system, and better advocates for themselves and others.

Each meeting and interaction presented an opportunity to build the team’s skills, confidence, and sense of belonging to the group. By listening to each interview as a group, we could all acknowledge the strengths in the way kin caregivers on the team asked questions and responded during often emotional interviews. One caregiver reflected on her growing confidence after a first pilot interview:

She was an angel sent just for me. … She was so excited that HALOS sent someone to call her that would understand, and she could talk to… I felt so good after I left the call because I helped her in a little way by just listening to her. … This is easy. I was so nervous from the beginning. … I thank you all for the confidence for building me up.

Another caregiver declared at the end of an interview: “I leave this interview, I feel changed.” At the end of the evaluation, one caregiver reflected, “I feel like you’ve given us wings.”

Since our evaluation, our team has remained active in HALOS. One kin caregiver on our team serves as a mentor to new kin caregivers. Another caregiver has been involved in training new staff to work effectively with kin caregivers and was invited to speak to the Governor of South Carolina about the needs of kinship families.

### Lessons learned #5: increasing the likelihood the nonprofit will act on the study findings

Ultimately, the purpose of the evaluation was to provide valid, culturally-responsive data to improve the services HALOS provides to kinship families. By involving stakeholders—both staff and program participants—in the evaluation process, who have a deep understanding of local context and culture, there is an expectation the findings will be more relevant, and thus, more useful [14, 15]. Even with the strengths of this approach, stakeholder participation and evaluation can fail to influence program decisions leaving stakeholders’ voices unheard [54, 55].

Many aspects of our process were intended to enhance HALOS’s appreciation for a participatory approach to learning. HALOS staff and kin caregivers participated in the evaluation training and planning together, so they were invested in the process and the outcome from the start. Throughout the analysis phase, we shared interview findings with the full HALOS team iteratively to ensure the information we were collecting would be valuable to them and to avoid any surprises. Finally, when we shared the final evaluation report with the full HALOS team, we shared questions for consideration rather than specific program recommendations. That way, we could meet with program staff to talk through potential program changes together and position ourselves as colleagues all working toward the same goal of improving services for kinship families.

Together, these steps enhanced HALOS’s orientation to act on the evaluation findings. Following our evaluation, HALOS instituted several program changes suggested through the evaluation process. For example, HALOS created an introductory packet to ensure caregivers understand all program offerings to which they would have access and developed a peer mentoring program to ensure new kin caregivers have the benefit of connecting with more experienced caregivers. Also, HALOS now includes our full evaluation report in staff training materials.

After completing the evaluation, HALOS began investing more effort in gathering data from caregivers to inform programming. Specifically, HALOS began taking greater steps to boost the caregiver response rate to the data collection that occurs at the end of the 3-month program by adding a $10 incentive and increasing outreach. In the 6 months leading up to this evaluation the response rate to the 3-month follow-up was 32%; In the 6 months following the evaluation, the response rate rose to 73%. Strategies to increase the response rate are particularly important to the kinship care field, as studies have discussed the difficulty of reaching informal kin caregivers and understanding their needs [56].

## Limitations

As a formative evaluation effort there are some limitations to note. We recognize the limits of understanding the lives of kinship caregivers through a single, retrospective interview and a pre-post needs assessment of a single group. In an observational study, it is not possible to isolate the impact of the program. Rather, this formative evaluation effort was designed to strengthen the HALOS program model and improve service delivery. Of note, of the 146 kin caregivers who entered the program, 46 completed both the pre- and post-Family Resource Scale. Further, our team interviewed just 31 of the 146 kin caregivers who entered the program in the study year. While interviewees and survey respondents had similar demographic characteristics to the full population of HALOS caregivers, their lives and experiences may be different in important ways.

One of the strengths of this evaluation was that data collection and analysis was led by kin caregivers who intimately understood kinship care. We recognize, too, that this approach had potential limitations. It may be that some interviewees were hesitant to share experiences that would put them in a negative light with their peers due to a social desirability bias. Interviewees may have been fearful of sharing negative feedback with our evaluation team, worried it may limit future program benefits. To help allay this fear, both during our consent process and at the beginning of each interview, we assured interviewees that their responses would be kept confidential and that we would share feedback only in summary form, without caregiver names.

We recognize, too, that there are both risks and benefits of including program staff on the evaluation team. We understood that kin caregivers may be less critical in their analysis of the program and in their program recommendations in an effort to avoid offending program staff. While it may be that excluding program staff would eliminate these concerns, we believed that the potential benefits would outweigh the potential risks. By having program staff involved, we had a better understanding of the nuances of the program model, could communicate our progress and emerging findings easily throughout the study, and were motivated to continue seeing how the findings would be of value to the program. To lessen the potential risks, the Kinship Director stepped away from all meetings involving analysis of interview data to ensure kin caregivers could listen and reflect on interviews freely. Program staff repeatedly conveyed to kin caregivers on the evaluation team that their critiques were necessary to further improve the program.

## Conclusion

This article demonstrates the value of involving people with lived experience at every stage of a program evaluation. Ensuring that a team of Black kinship caregivers had decision-making power at every stage of the evaluation was an important move towards racially equitable evaluation methods. Through this process, we learned that participatory evaluation is a feasible and useful approach both to understanding the experiences of kinship families and to improving the supports in their lives.

We hope this paper will inspire others to draw on the strengths and capacity of people with lived experience to engage in participatory evaluation. Greater recognition of the value of this approach in social change and increased funding to carry out the process are both needed [21, 46]. The lessons learned apply to kinship care but also to a broad array of fields in which participants could be more widely embraced in formative evaluations of social programs.

## Data Availability

Not applicable.

## Abbreviations

Kin: Kinship
U.S.: United States
